# The effect of endothelial nitric oxide synthase on the hemodynamics and wall mechanics in murine arteriovenous fistulas

**DOI:** 10.1038/s41598-019-40683-7

**Published:** 2019-03-12

**Authors:** Daniel Pike, Yan-Ting Shiu, Yun-Fang Cho, Ha Le, Maheshika Somarathna, Tatyana Isayeva, Lingling Guo, J. David Symons, Christopher G. Kevil, John Totenhagen, Timmy Lee

**Affiliations:** 1 Department of Biomedical Engineering, University of Utah, Salt Lake City, UT USA; 2 Division of Nephrology and Hypertension, Department of Internal Medicine, University of Utah, Salt Lake City, UT USA; 3 Veterans Affairs Medical Center, Salt Lake City, UT USA; 4 Department of Medicine and Division of Nephrology, University of Alabama at Birmingham, Birmingham, AL USA; 5 Department of Nutrition and Integrative Physiology and Molecular Medicine Program, University of Utah, Salt Lake City, UT USA; 6 Division of Endocrinology, Metabolism, and Diabetes, University of Utah, Salt Lake City, UT USA; 7 Departments of Pathology, Molecular and Cellular Physiology, and Cellular Biology and Anatomy, LSU Health Shreveport, Shreveport, LA USA; 8 Department of Radiology, University of Alabama at Birmingham, Birmingham, AL USA; 9 Veterans Affairs Medical Center, Birmingham, AL USA

**Keywords:** 3-D reconstruction, Haemodialysis

## Abstract

Creation of a hemodialysis arteriovenous fistula (AVF) causes aberrant vascular mechanics at and near the AVF anastomosis. When inadequately regulated, these aberrant mechanical factors may impede AVF lumen expansion to cause AVF maturation failure, a significant clinical problem with no effective treatments. The endothelial nitric oxide synthase (NOS3) system is crucial for vascular health and function, but its effect on AVF maturation has not been fully characterized. We hypothesize that NOS3 promotes AVF maturation by regulating local vascular mechanics following AVF creation. Here we report the first MRI-based fluid-structure interaction (FSI) study in a murine AVF model using three mouse strains: NOS3 overexpression (NOS3 OE) and knockout (NOS3−/−) on C57BL/6 background, with C57BL/6 as the wild-type control (NOS3+/+). When compared to NOS3+/+ and NOS3−/−, AVFs in the OE mice had larger lumen area. AVFs in the OE mice also had smoother blood flow streamlines, as well as lower blood shear stress at the wall, blood vorticity, inner wall circumferential stretch, and radial wall thinning at the anastomosis. Our results demonstrate that overexpression of NOS3 resulted in distinct hemodynamic and wall mechanical profiles associated with favorable AVF remodeling. Enhancing NOS3 expression may be a potential therapeutic approach for promoting AVF maturation.

## Introduction

The arteriovenous fistula (AVF), created by a direct anastomosis between a peripheral artery and vein, is the preferred choice of vascular access for hemodialysis patients. However, AVF maturation failure remains a critically important clinical problem and currently there are no effective treatments to promote AVF maturation^1–3^. AVF maturation failure results in dialysis therapy with other types of vascular access including a tunneled dialysis catheter or a synthetic arteriovenous graft (AVG). Mortality rates in patients dialyzing with catheters have been reported to be 1.4 and 1.1 times greater than that in patients dialyzing with AVFs and AVGs, respectively^4^. If *successfully matured* for dialysis, an AVF is favored over an AVG due to its higher long-term patency and lower long-term intervention rates^5^. Further, compared to patients dialyzing with a mature AVF, the relative mortality risk has been reported as 1.4 fold higher for patients using an AVG^6^.

Currently up to 60% of newly created AVFs do not successfully mature to become usable for hemodialysis^7–10^. The two main etiologies of AVF maturation failure are aggressive neointimal hyperplasia (NH) development and inadequate lumen expansion of the AVF vein^11,12^. NH, which usually occurs at and near the AVF anastomosis, is primarily a result of vascular smooth muscle cells (SMCs) and fibroblasts migrating and proliferating from the vessel media into the intima^11,13^. Lumen expansion involves the relaxation of vascular SMCs upon stimulation by vasodilators released from endothelial cells (ECs)^14–17^. The shunting of arterial flow into the vein decreases the resistance and thus causes the regional AVF flow to be 5- to 10-fold higher than normal arterial flow in patients^18,19^. Such a drastic increase in volumetric blood flow, coupled with the acute change in blood flow direction at the anastomosis, causes drastically aberrant vascular mechanics (i.e., hemodynamics and wall mechanics) in the anastomotic region. Based on the wealth of literature regarding the effects of hemodynamics and wall mechanics on arterial wall function and remodeling, it has long been postulated that aberrant vascular mechanics may lead to NH formation and/or inadequate lumen dilation, and ultimately AVF maturation failure. However, whether these relationships exist in the vein are not yet clear, as venous ECs and SMCs are known to have different phenotypes from their arterial counterparts^20–22^. Importantly, detailed hemodynamics and wall mechanics in the AVF are not yet fully available.

Nitric oxide (NO) has been well-established as beneficial to vascular health and function^23,24^. NO derived from endothelial nitric oxide synthase (eNOS, also known as NOS3) stimulates arterial^17^ and venous^16,17^ vasorelaxation via increasing the generation of cyclic guanosine monophosphate (cGMP) to cause active smooth muscle relaxation, and inhibits arterial SMC migration and proliferation^25,26^, all of which could lead to favorable AVF development. However, the effect of NOS3 on AVF remodeling has not been fully characterized. In addition, whether its effect is mediated by vascular mechanics of the AVF remains unknown. The present study aims to address these questions by investigating AVF remodeling and characterizing vascular mechanics in three mouse strains with different NOS3 expression levels. To do so, we have made significant contributions to the development of image-based biomechanical modeling tools for understanding the complex hemodynamics in AVFs. We first developed an MRI-based computational fluid dynamics (CFD) method that can be used on AVFs in hemodialysis patients^27^. Next, we refined the MRI-based CFD to characterize the hemodynamics of murine AVFs at very high temporal and spatial resolutions^28^. These two previous studies assumed the blood vessel wall to be rigid. Here we present the first MRI-based fluid-structure interaction (FSI) pipeline to characterize *both hemodynamics and wall mechanics* in murine AVFs with varying NOS3 expression levels in *deformable* blood vessel walls.

## Results

A photograph of a representative mouse AVF is shown in Supplementary Fig. S1. Our experience is that NH in the wild-type control was significant at Week 3. Therefore, we investigated AVF lumen and NH (Fig. 1) at 21 days after AVF creation in mice with varying NOS3 expression levels, as well as their hemodynamics (Figs 2–5) and wall mechanics (Figs 6–8).

**Figure 1:**
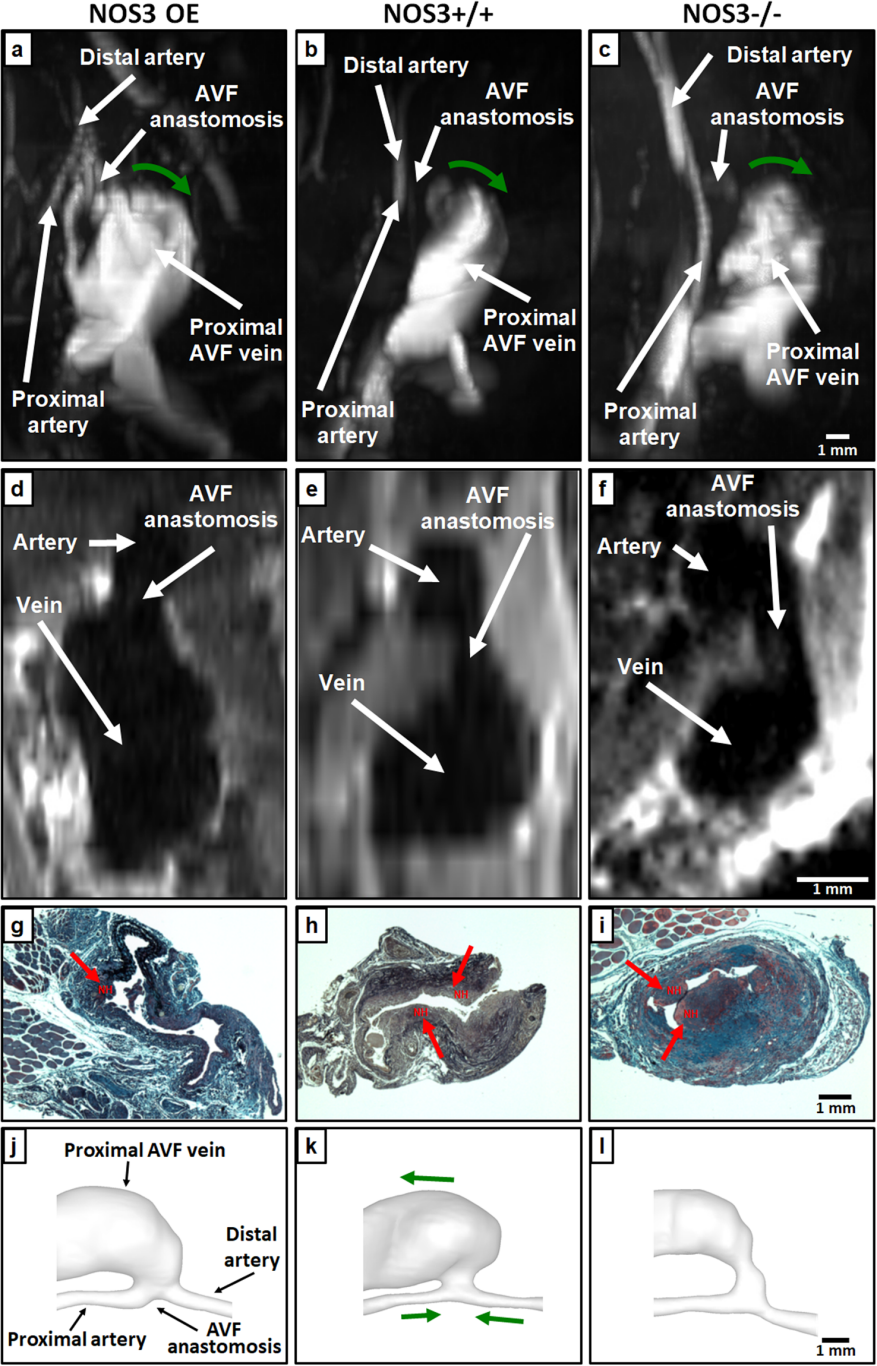
MRI images, histological images and lumen geometrical models at 21 days after AVF creation. 2D time of flight MRI images of NOS3 OE (**a**) NOS3+/+ (**b**) and NOS3−/− (**c**) mice. Green arrows indicate the direction of blood flow in the proximal AVF vein. Scale bar in (**c**) also applies to (**a**) and (**b**) 3D multiplanar black-blood MRI images of NOS3 OE (**d**), NOS3+/+ (**e**) and NOS3−/− (**f**) mice. Scale bar in (**f)** also applies to (**d**) and (**e**) Russell-Movat pentachrome-stained proximal AVF vein histological images for NOS3 OE (**g**), NOS3+/+ (**h**), and NOS3−/− (**i**) mice. Red arrows point to the venous neointimal hyperplasia (NH). Scale bar in (**i**) also applies to (**g**) and (**h**). The lumen geometrical models of the AVFs of NOS3 OE (**j**), NOS3+/+ (**k**) and NOS3−/− (**l**) mice for simulations. The labeling of blood vessels in (**j**) the green arrows in (**k**) (which indicate the direction of blood flow) and the scale bar in (**l**) applies to all 3 lumen geometrical models. Note the presence of backflow in the distal artery in all three mice, which is the same to what we observed in AVFs in hemodialysis patients^27^.

**Figure 2:**
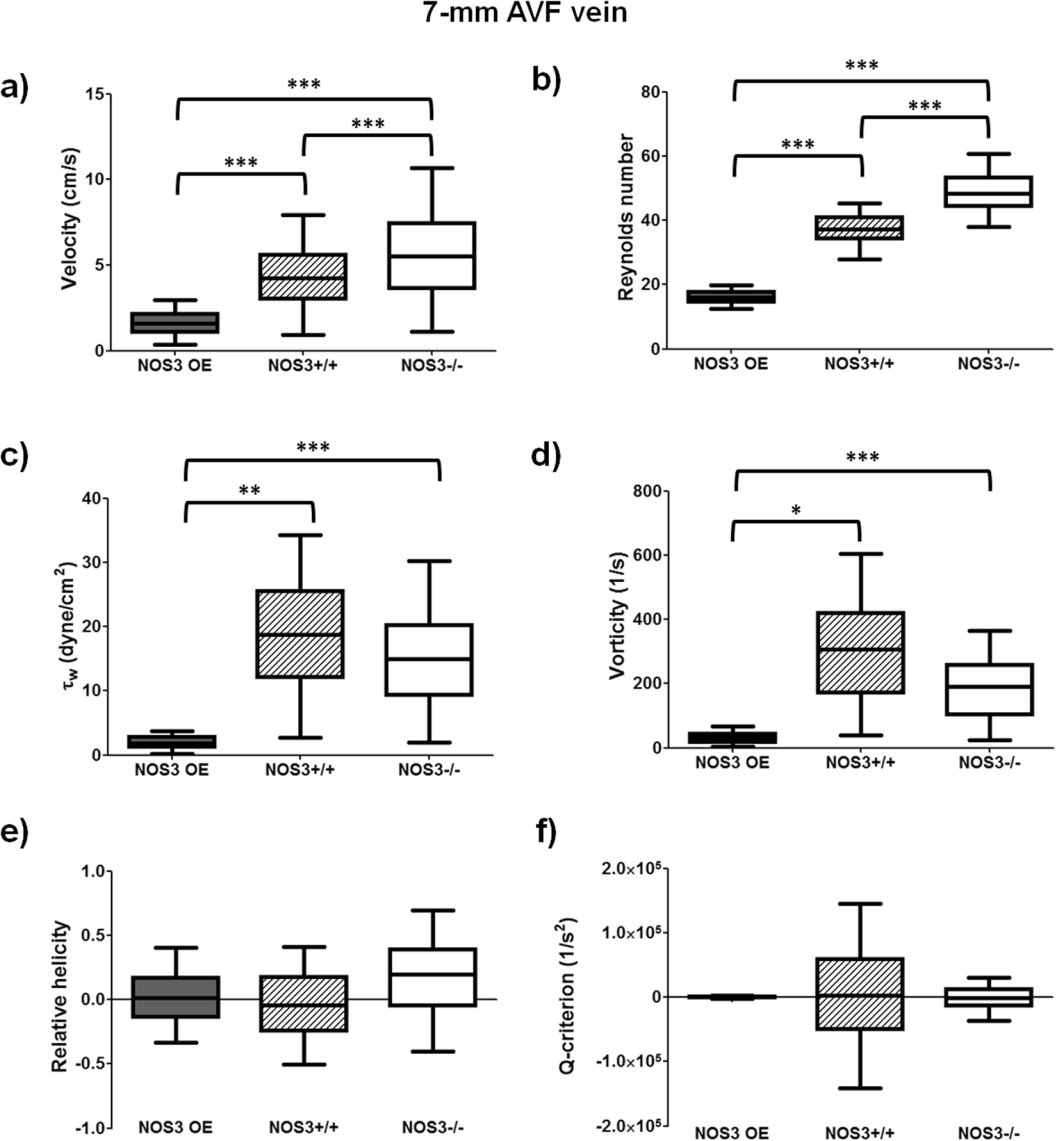
Averaged AVF hemodynamics at 21 days after creation. Box plots of velocity (**a**) Reynolds number (**b**) fluid shear stress at the AVF wall (i.e., τ_w_) (**c**), vorticity (**d**), relative helicity (**e**), and Q-criterion (**f**) were averaged over both a cardiac cycle and the first 7 mm of the proximal AVF vein starting from the anastomosis (140 cross-sectional slices, with 50 μm between 2 slices) in the lumen geometrical models shown in Fig. 1(j–l). Box plots show 25th to 75th percentile, with whiskers of 5% and 95% of data range. *p < 0.05; **p < 0.01; ***p < 0.001. Baseline (i.e., pre-surgery jugular vein in NOS3+/+ mice) values were: velocity = 0.04 ± 0.02 cm/s; Reynolds number = 0.2 ± 0.1; τ_w_ = 1.0 ± 0.3 dyne/cm^2^; vorticity = 20.4 ± 10.3 1/s; relative helicity = 0.005 ± 0.03; Q-criterion = 1.3 ± 18.7 1/s^2^.

**Figure 3:**
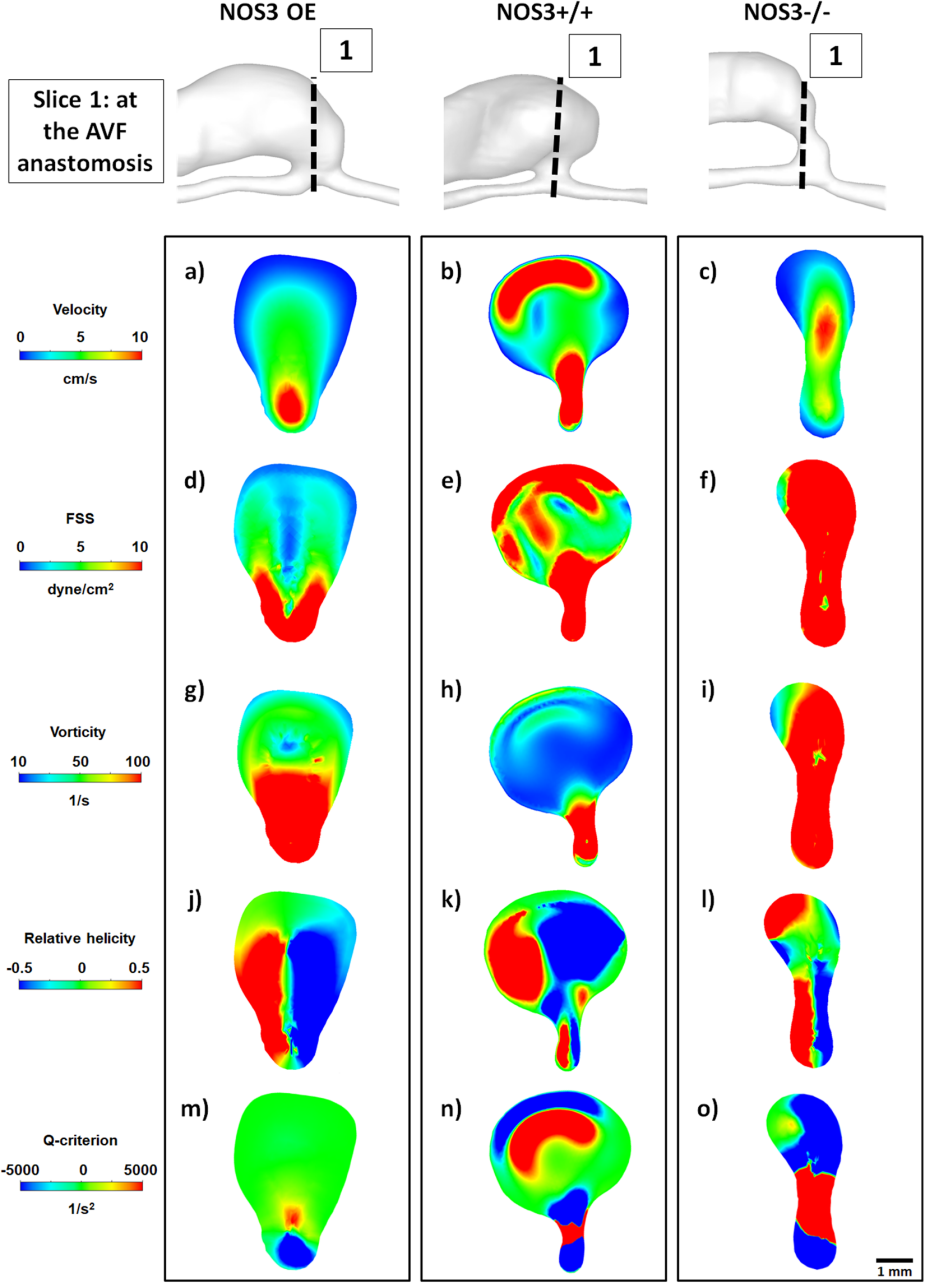
Hemodynamics at the AVF anastomosis at 21 days after creation. Color maps of the velocity (1^st^ panel), luminal and wall fluid shear stress (FSS) (2^nd^ panel), vorticity (3^rd^ panel), relative helicity (4^th^ panel) and Q-criterion (5^th^ panel) at Slice 1 (indicated by the dashed black line in the lumen geometrical models), which is a cross-section located at the AVF anastomosis, during systole. Scale bar in (**o**) applies to (**a–o**). Color scales are set to best contrast the differences among the three mice.

**Figure 4:**
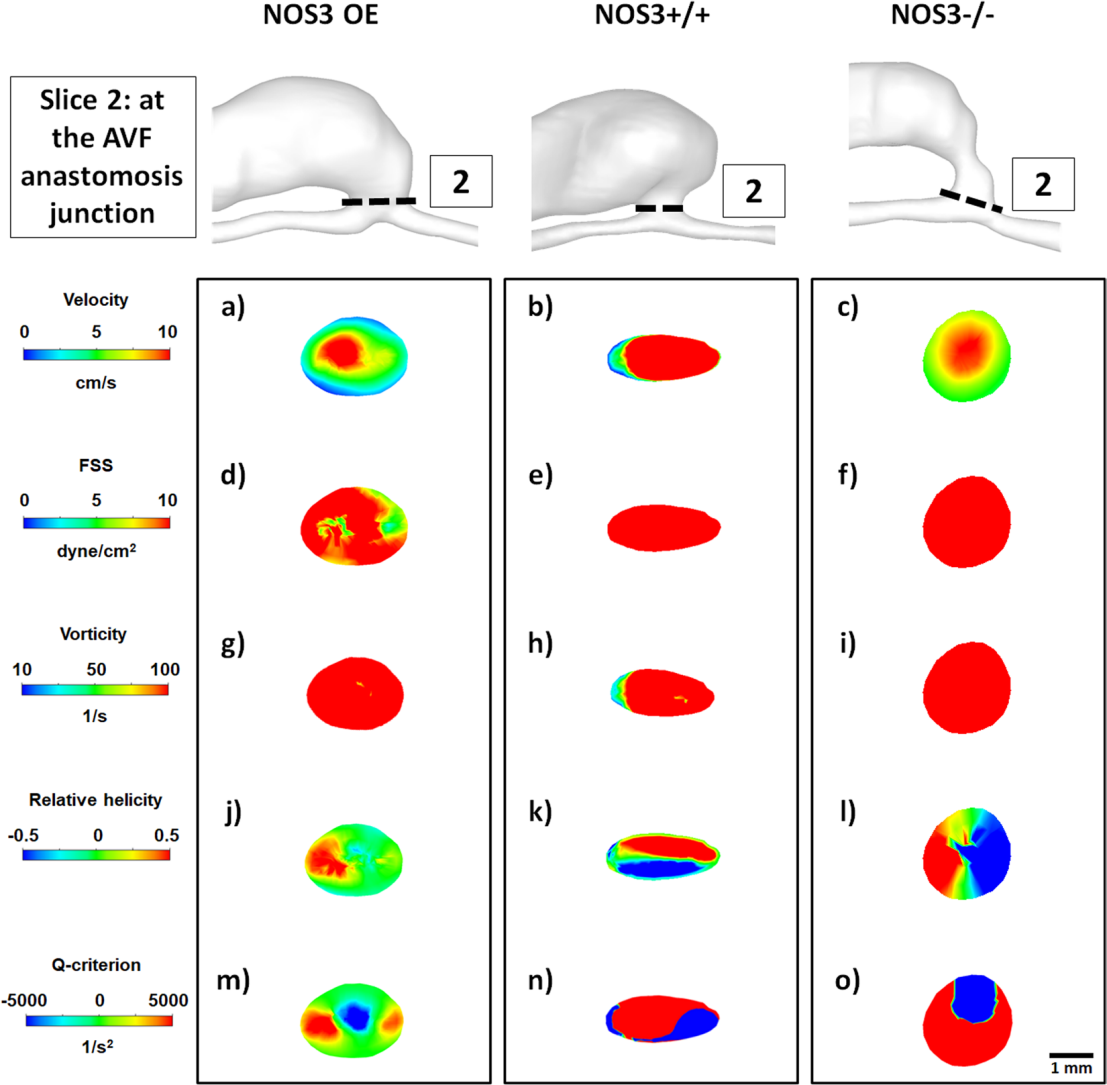
Hemodynamics at the AVF anastomosis junction at 21 days after creation. Color maps of the velocity (1^st^ panel), luminal and wall fluid shear stress (FSS) (2^nd^ panel), vorticity (3^rd^ panel), relative helicity (4^th^ panel) and Q-criterion (5^th^ panel) at Slice 2 (indicated by the dashed black line in the lumen geometrical models), which is a cross-section located at the AVF anastomosis junction where sutures were placed, during systole. Scale bar in (**o**) applies to (**a–o**). Color scales are set to best contrast the differences among the three mice.

**Figure 5:**
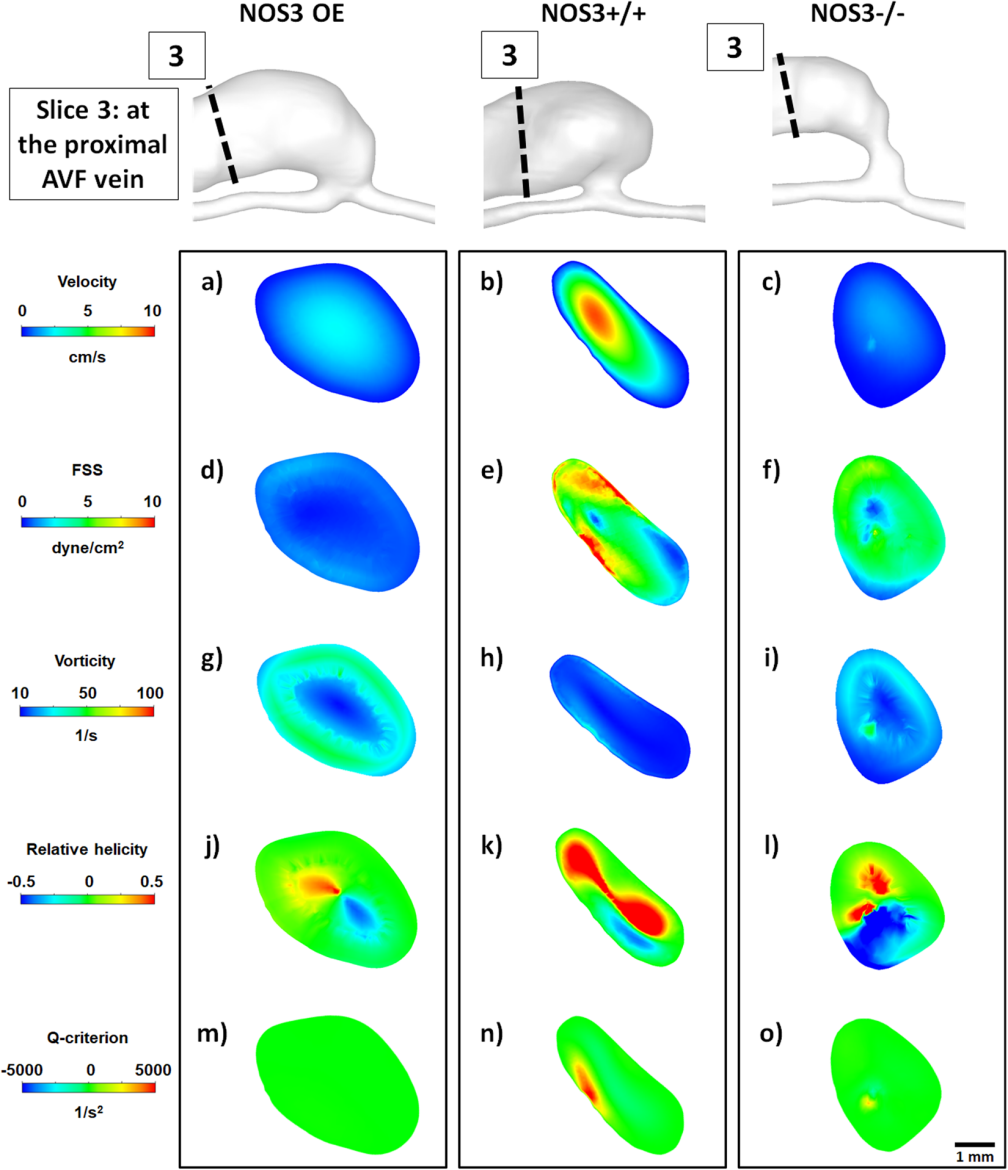
Hemodynamics at the proximal AVF vein at 21 days after creation. Color maps of the velocity (1^st^ panel), luminal and wall fluid shear stress (FSS) (2^nd^ panel), vorticity (3^rd^ panel), relative helicity (4^th^ panel) and Q-criterion (5^th^ panel) at Slice 3 (indicated by the dashed black line in the lumen geometrical models), which is a cross-section located at the proximal AVF vein, during systole. Scale bar in (**o**) applies to (**a–o**). Color scales are set to best contrast the differences among the three mice.

**Figure 6:**
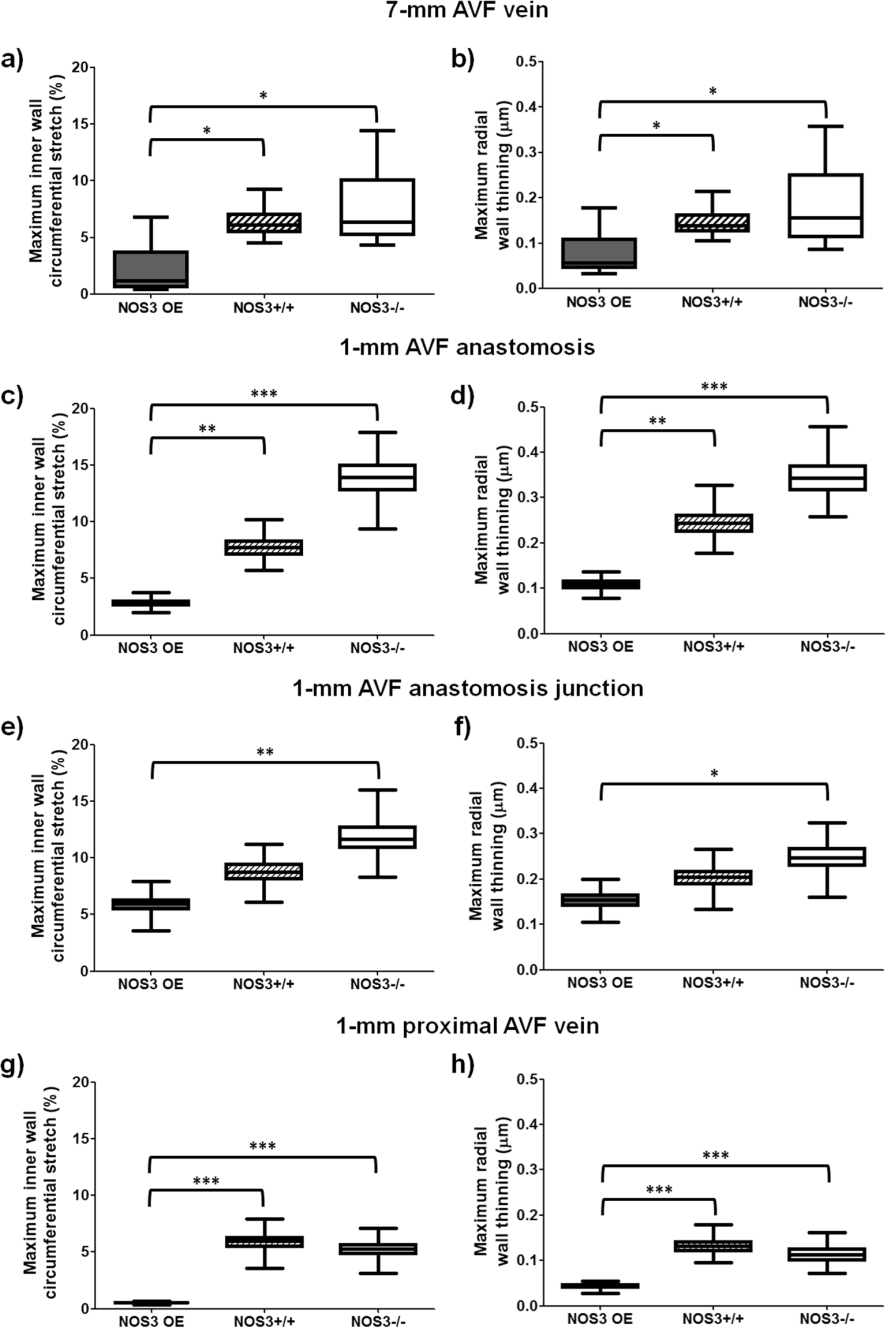
Averaged AVF inner wall circumferential stretch and radial wall thinning at 21 days after creation. The maximum inner wall circumferential stretch within a cardiac cycle at each cross-sectional slice was averaged over either 7 or 1 mm at the indicated region. The radial wall thinning was averaged around the circumference of each slice first; next, the maximum radial wall thinning within a cardiac cycle at each slice was averaged over either 7 or 1 mm at the indicated region. The distance between 2 slices is 50 μm. Box plots of the maximum inner wall circumferential stretch of the 7-mm AVF (**a**), 1-mm AVF anastomosis (**c**), 1-mm AVF anastomosis junction (**e**) and 1-mm proximal AVF vein (**g**). Box plots of maximum radial wall thinning of the 7-mm AVF (**b**), 1-mm AVF anastomosis (**d**), 1-mm AVF anastomosis junction (**f**) and 1-mm proximal AVF vein (**h**). Box plots show 25th to 75th percentile, with whiskers of 5% and 95% of data range. *p < 0.05; **p < 0.01; ***p < 0.001. Baseline (i.e., pre-surgery jugular vein in NOS3+/+ mice) values were: maximum inner wall circumferential stretch = 0.02 ± 0.004%; maximum radial wall thinning = 0.01 ± 0.003 μm.

**Figure 7:**
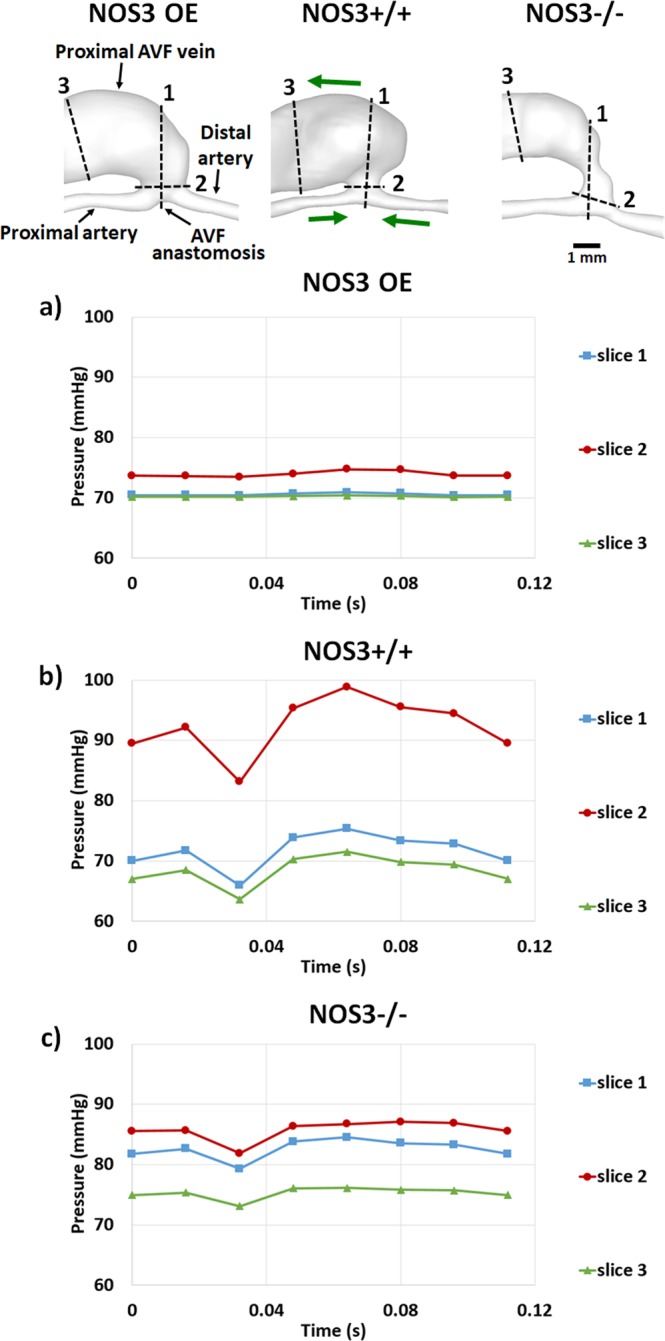
AVF luminal pressure over the cardiac cycle from the FSI simulations of NOS3 OE (**a**), NOS3+/+ (**b**), and NOS3−/− (**c**) mice. In the top panel, the labeling of the blood vessels in NOS3 OE, green arrows in NOS3+/+ (which indicate the direction of blood flow) and the scale bar in NOS3−/− apply to all three mice. Pressure is averaged over the cross-sectional area of each slice indicated in the top panel: Slice 1 is located at the AVF anastomosis, Slice 2 is located at the AVF anastomosis junction, and Slice 3 is located at the proximal AVF vein.

**Figure 8:**
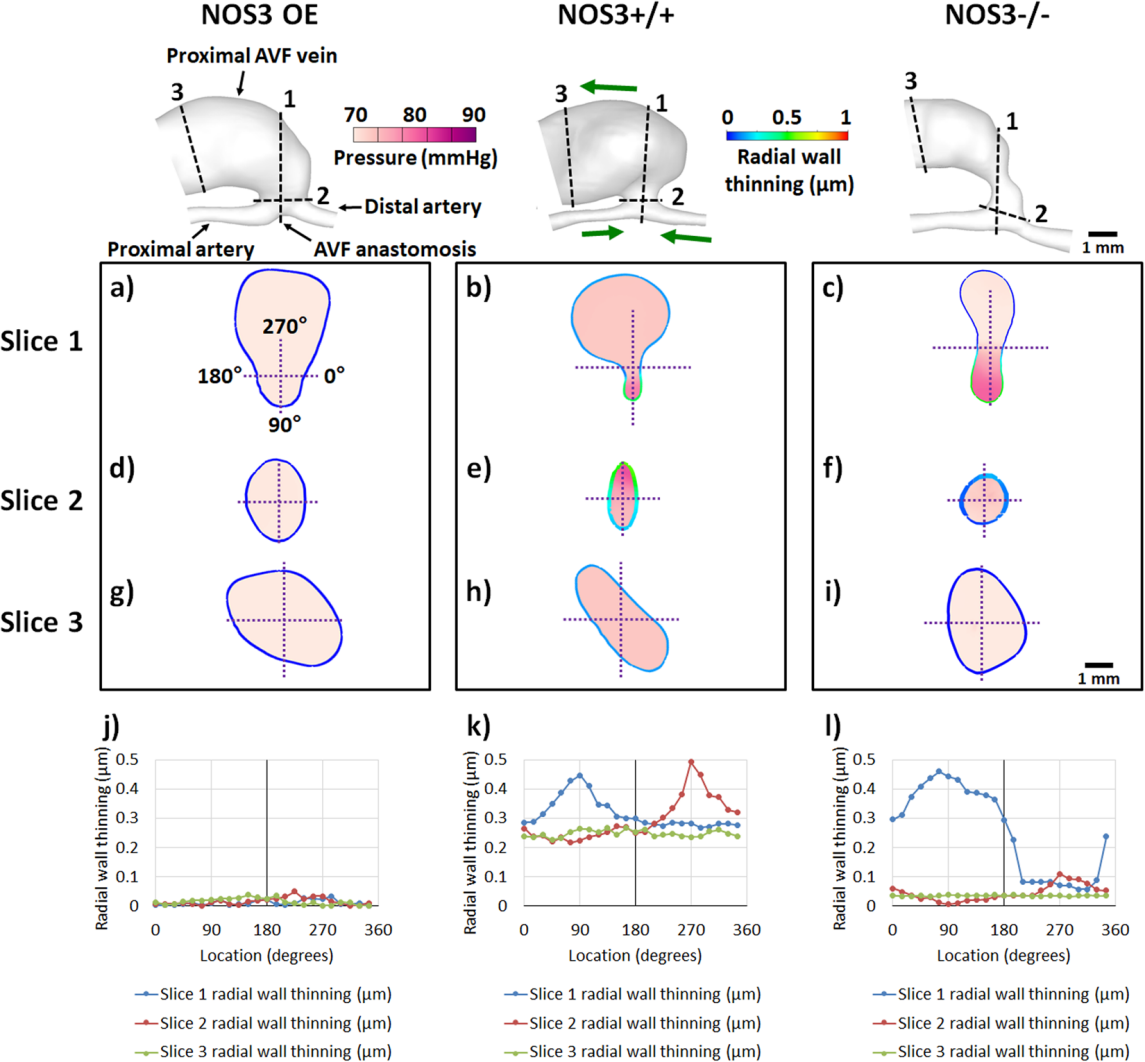
Radial wall thinning and luminal pressure at the AVF anastomosis, AVF anastomosis junction, and proximal AVF vein at 21 days after creation. In the top panel, the labeling of the blood vessels in NOS3 OE, green arrows in NOS3+/+ (which indicate the direction of blood flow) and the scale bar in NOS3−/− apply to all three mice. In (**a**–**i**), the lumen of cross-sections at Slices 1 (located at the AVF anastomosis), 2 (located at the AVF anastomosis junction), and 3 (located at proximal AVF vein) is filled with a color map of luminal pressure, and the wall is colored with the magnitude of radial wall thinning, both during systole. The coordinates in (**a**) and the scale bar in (**i**) apply to (**a**–**i**). In (**j**–**l**), line charts of radial wall thinning during systole are graphed along the circumference of the wall, using the coordinates shown in (**a**).

### Mouse AVF lumen geometry by MRI and NH by histology

Two-dimensional (2D) time-of-flight (ToF) MRI was used to visualize the global lumen geometry of the AVF. This is a common non-contrast-agent bright-blood method for imaging the vasculature, where the flowing blood (hence lumen) appears white. Figure 1a–c shows maximum intensity projection (MIP) images from the 2D ToF MRI for all three mouse strains; the NOS3 OE mouse had larger overall AVF lumen than NOS3+/+ and NOS3−/− mice. A different type of MRI scan with higher spatial resolution was then performed to reveal the AVF lumen, particularly the anastomosis, more clearly to create lumen geometrical models for simulations. Figure 1d–f shows the black-blood (BB) MRI image of lumen at the AVF anastomosis. BB MRI registers a lack of signal (black) when blood is flowing through the slice of interest, thus black indicates the vessel lumen. White signals in BB MRI images indicate static tissue. As shown in Fig. 1d, the NOS3 OE mouse had a clearly defined and open anastomosis connecting the artery and the expanded proximal AVF vein. In both of the NOS+/+ and NOS3−/− mice (Fig. 1e,f), the anastomosis appeared to be narrow; the proximal AVF vein was not as large as in the NOS3 OE mouse, suggesting more pronounced NH formation and/or impaired lumen expansion in the NOS3+/+ and NOS3−/− mice. Indeed, NH in the NOS3 OE mouse was small, and the NOS3+/+ and NOS3−/− mice had extensive NH, as seen in the Russell-Movat pentachrome-stained histological images of the proximal AVF vein cross-sections (Fig. 1g–i).

The BB images were used for 3D reconstructions of the AVF lumen, which were then used to build lumen geometrical models for the FSI simulations. Figure 1j–l shows the 3D reconstructions of the AVF lumen from NOS3 OE, NOS3+/+ and NOS3−/− mice. We calculated the lumen cross-sectional area in the proximal AVF vein using these 3D reconstructions. The AVF lumen cross-sectional area, which was averaged over 7 mm starting from the anastomosis, was significantly larger in the NOS3 OE mouse (7.1 ± 1.5 × 10^6^ µm^2^) as compared to the NOS3+/+ mouse (5.0 ± 1.2 × 10^6^ µm^2^, p < 0.01) and the NOS3−/− mouse (2.2 ± 1.1 × 10^6^ µm^2^, p < 0.01); however, the AVF lumen of all mice was bigger than the lumen of the baseline, wild-type presurgical jugular vein (0.6 ± 0.2 × 10^6^ µm^2^, p < 0.01).

Taken together, our MRI images, histological images and 3D reconstructions show that the NOS3 OE mouse had the most favorable AVF remodeling, with the biggest lumen expansion and smallest NH. We next performed FSI simulations to characterize the hemodynamics and wall mechanics of the AVFs.

### Mouse AVF hemodynamics by FSI

Figure 2a–c show the velocity, Reynolds number (a unitless measure of blood flow turbulence), and the wall hemodynamic parameter τ_w_ (which is the fluid shear stress at the wall, i.e., the frictional forces between the flowing blood and the vessel wall). Figure 2d–f show volumetric hemodynamic parameters (i.e., vorticity, relative helicity and Q-criterion) that can be used to identify a vortex. Vorticity is a measurement of the magnitude of blood rotation with no directional component. Relative helicity is a normalized measurement of the direction of blood rotation ranging from +1 (completely clockwise) to −1 (completely counterclockwise). Q-criterion is a measurement of the dominance of parallel blood flow (e.g., in the direction of bulk flow) or rotational blood flow; a positive Q-criterion value indicates dominant rotational blood flow and a possible vortex, whereas a negative Q-criterion value indicates dominant parallel flow with little rotation and no vortex. These six hemodynamic parameters were averaged over both a cardiac cycle and the first 7 mm of the AVF vein starting from the anastomosis (140 cross-sectional slices, with 50 μm between 2 slices) in NOS3 OE, NOS3+/+ and NOS3−/− mice at 21 days after AVF creation. The NOS3 OE mouse had lower blood flow velocity (Fig. 2a) than the NOS3+/+ (p < 0.001) and NOS3−/− (p < 0.001) mice. Although the Reynolds number (Fig. 2b) was well below the threshold for turbulent flow (which is 400–550^29^) in all strains, it was smaller in the NOS3 OE mice than in the NOS3+/+ (p < 0.001) and NOS3−/− (p < 0.001) mice. Indeed, the blood velocity streamlines in the NOS3 OE mice were smoother than those in the NOS3+/+ and NOS3−/− mice (Supplementary Fig. S2). Furthermore, the NOS3 OE mouse had significantly lower τ_w_ (Fig. 2c) and vorticity (Fig. 2d) than the NOS3+/+ (τ_w_, p < 0.01; vorticity, p < 0.05) and NOS3−/− (τ_w_, p < 0.001; vorticity, p < 0.001) mice, suggesting that lower τ_w_ and vorticity were associated with favorable AVF remodeling. Importantly, τ_w_ in the NOS3 OE mouse (2.1 ± 1.5 dyne/cm^2^) was the closest to τ_w_ in our baseline, wild-type presurgical mouse jugular vein, which was 1.0 ± 0.3 dyne/cm^2^, indicating normalization (i.e., return to baseline) of flow in the AVF with favorable remodeling. There was no statistically significant difference in relative helicity (Fig. 2e) and Q-criterion (Fig. 2f) among these mouse strains. Finally, we also calculated the oscillatory shear index at the wall (OSI), which describes the magnitude of change in the blood flow direction over a cardiac cycle and it value ranges between 0 and 0.5. Although large OSI has been associated with pathological arterial remodeling^30–32^, the OSI values in our murine AVFs were low (<0.001) in all three mice and there were no significant differences among them (data not shown).

Because aggressive NH development often occurs at and near the anastomosis, we next investigated the hemodynamic profile of this region (Figs 3 and 4) and compared it to the hemodynamics displayed by the proximal AVF vein (Fig. 5), which is less prone to NH.

Figure 3 shows color maps of velocity and hemodynamic parameters, including both luminal and wall fluid shear stress (FSS), vorticity, relative helicity, and Q-criterion, in a cross-section located *at the AVF anastomosis*. While all averaged values (i.e., averaged over both a cardiac cycle and cross-sectional area) are smaller in NOS3 OE mice when compared to NOS3+/+ and NOS3−/− mice, it is important to point out that mice with different NOS3 expression levels had distinctly and qualitatively different hemodynamic patterns. The NOS3 OE mouse had higher velocity at the arterial side of the anastomosis, which gradually transitioned to lower velocity at the venous side; this pattern was also observed for FSS and vorticity. In contrast, this smooth transition was not seen in the NOS3+/+ and NOS3−/− mice. Further, there were only two symmetrical areas of relative helicity in the NOS3 OE mouse (one positive, the other negative), suggesting a smooth change in the direction of blood flow from the arterial to the venous side. In contrast, there were several areas of both positive and negative relative helicity in the NOS3+/+ and NOS3−/− mice, suggesting chaotic, disturbed flow. Regions of positive Q-criterion (indicating dominant rotational flow) in the NOS3 OE mouse were small and localized at the arterial side of the anastomosis, while regions of positive Q-criterion in the NOS3+/+ and NOS3−/− mice were larger and present in both the arterial and venous sides of the anastomosis.

Figure 4 shows color maps of velocity and hemodynamic parameters in a cross-section located *at the AVF anastomosis junction*, where sutures were placed during the AVF creation surgery. Again, mice with different NOS3 expression levels had distinctly and qualitatively different hemodynamic patterns. In the NOS3 OE, velocity was the largest in the lumen center and became smaller gradually and symmetrically toward the wall and zero at the wall, suggesting a fully developed laminar flow pattern. In contrast, velocity in NOS3+/+ and NOS3−/− mice was high throughout most of the cross-section including the wall. Accordingly, the size of helical flow regions (as measured by positive or negative relative helicity) and dominant rotational flow regions (as measured by positive Q-criterion) were smaller in the NOS3 OE mouse than in the NOS3+/+ and NOS3−/− mice. Finally, while luminal and wall FSS in the NOS3 OE was ≤10 dyne/cm^2^ and FSS in NOS3+/+ and NOS3−/− mice was ≥10 dyne/cm^2^ throughout the cross-section, vorticity was similar in all three mice at ≥50 1/s.

Figure 5 shows color maps of velocity and hemodynamic parameters in a cross-section located *at the proximal AVF vein*, which is 5 mm downstream from the AVF anastomosis junction. All values are generally smaller when compared to those at the AVF anastomosis junction, which is likely due to the bigger lumen area at this location than at the AVF anastomosis junction. The averaged velocity, luminal and wall FSS, and Q-criterion in the NOS3 OE mouse were significantly smaller than those in the NOS3+/+ and NOS3−/− mice. Although vorticity was higher in the NOS3 OE and NOS3−/− mice (≤50 1/s) than in the NOS3+/+ mouse (≤20 1/s), the averaged values for all three mouse strains were smaller than that in the AVF anastomosis (Fig. 3g–i) and the AVF anastomosis junction (Fig. 4g–i). Relative helicity patterns were symmetrical with two small and symmetrical regions (one positive, the other negative) of relative helicity in the NOS3 OE mouse, but the relative helicity regions were bigger and more asymmetrical in the NOS3+/+ and NOS3−/− mice. Positive Q-criterion regions were present in the NOS3+/+ and NOS3−/− mice but not the NOS3 OE mouse.

### Mouse AVF wall mechanics by FSI

The baseline, wild-type presurgical jugular vein had negligible inner wall circumferential stretch (0.02 ± 0.004%) and radial wall thinning (0.01 ± 0.003 μm). Figure 6 shows the maximum inner wall circumferential stretch and the maximum radial wall thinning, which are averaged over both a cardiac cycle and 7 mm in the AVF vein starting from the anastomosis (140 slices, with 50 μm between 2 slices) or over a cardiac cycle and 1 mm length (20 slices, with 50 μm between 2 slices) at three locations: the AVF anastomosis, AVF anastomosis junction (where sutures were placed), and the proximal AVF vein. Within the 7-mm AVF vein (Fig. 6a,b), the NOS3 OE mouse had lower maximum inner wall circumferential stretch and maximum radial wall thinning when compared to both NOS3+/+ (stretch and thinning, p < 0.05) and NOS3−/− (stretch and thinning, p < 0.05) mice, suggesting that lower stretch and thinning were associated with favorable AVF remodeling. We observe the same patterns when taking a closer look at different regions. At the AVF anastomosis (Fig. 6c,d), the NOS3 OE mouse had lower stretch and thinning when compared to both NOS3+/+ (stretch and thinning, p < 0.01) and NOS3−/− (stretch and thinning, p < 0.001) mice. At the AVF anastomosis junction (Fig. 6e,f), the maximum inner wall circumferential stretch and maximum radial wall thinning were smaller in NOS3 OE than NOS3−/− (p < 0.01 and p < 0.05, respectively). In the proximal AVF vein (Fig. 6g,h), maximum inner wall circumferential stretch and maximum radial wall thinning were smaller in NOS3 OE than both NOS3+/+ and NOS3−/− (p < 0.001 for all comparisons).

Pressure is a critical factor driving vessel wall deformation. We found that the luminal pressure (Fig. 7a) remained low and uniform at approximately 70–73 mmHg over the cardiac cycle at all three locations in the NOS3 OE mouse: the AVF anastomosis (slice 1), AVF anastomosis junction (slice 2) and the proximal AVF vein (slice 3). In contrast, both luminal pressure magnitude and pulsatility were higher in the NOS3+/+ (Fig. 7b) and NOS3−/− (Fig. 7c) mice at the AVF anastomosis (slice 1) and AVF anastomosis junction (slice 2). The results of pressure are consistent with the results of wall deformation.

Figure 8a–i shows color maps of luminal pressure and radial wall thinning in a cross-section at the three locations: the AVF anastomosis (slice 1), the AVF anastomosis junction (slice 2) and the proximal AVF vein (slice 3). Figure 8j–l shows line charts of the values of radial wall thinning along the vessel circumference. The NOS3 OE mouse had minimal radial wall thinning. In contrast, the radial wall thinning in NOS3+/+ and NOS3−/− mice was elevated and asymmetrical along the circumference; peaks of radial wall thinning coincided with local pressure peaks.

### Mouse AVF biological responses: cGMP levels and intimal hyperplasia

**Figure 9:**
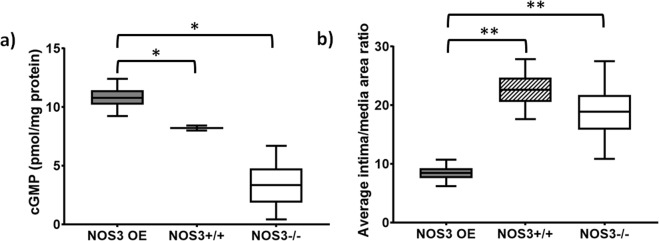
The levels of cGMP and the intima/media area ratios in the AVFs at 21 days after creation. (**a**) NOS3 OE mice had significantly increased cGMP levels when compared to NOS3+/+ and NOS3−/− mice. (**b**) NOS3 OE mice had significantly decreased intima/media area ratios when compared to NOS3+/+ and NOS3−/− mice. Data are expressed as mean± SEM. N = 3–4 per group. *Indicates p = 0.05 and **indicates p = 0.0005 when compared to NOS3+/+ and NOS3−/− by ANOVA.

Figure 9 shows the cGMP levels and intima/media area ratios in the AVFs of all three strains at Day 21. NOS3 OE mice had significantly increased cGMP levels when compared to both NOS3+/+ and NOS3−/− mice (Fig. 9a). Additionally, NOS3 OE mice had a significantly decreased intima/media area ratio compared to the other two strains (Fig. 9b). These results show that overexpression of NOS3 resulted in biological responses beneficial for AVF maturation, i.e., increasing cGMP to relax smooth muscle and decreasing NH formation to maintain a dilated lumen.

## Discussion

Our studies found that overexpression of NOS3 led to favorable AVF remodeling (i.e., bigger AVF lumen and smaller NH), smoother blood flow streamlines, as well as lower fluid shear stress at the wall, luminal fluid vorticity, circumferential inner wall stretch, and radial wall thinning. These results establish that NOS3 is a critical molecule driving successful AVF remodeling, and that the effect of NOS3 on AVF remodeling may be mediated, at least in part, through its effect on vascular mechanics (i.e., hemodynamics and wall mechanics).

The mechanisms leading to AVF maturation failure remain poorly understood, but are believed to be related to aggressive NH development and/or insufficient lumen expansion. NO has been well-established as beneficial to vascular health and function in the arterial vasculature^23,33^. Because the NOS3-derived NO from ECs stimulates vascular SMC relaxation (and hence vessel dilation) by activating guanylate cyclase to upregulate cGMP^15,34^ and inhibits vascular SMC migration and proliferation^24,25^, the NOS3-NO system is expected to be beneficial for AVF remodeling. Several publications have supported this notion. In the NO front, Tong *et al*. isolated SMCs from human vein samples used for creating AVFs in CKD patients and found that NO responsiveness of human venous SMCs *in vitro* was associated with AVF maturation *in vivo*^35^. Additionally, Geenen *et al*. showed that rats with 5/6 nephrectomy to induce CKD had impaired AVF remodeling and NO-mediated vasodilation, and that administration of soluble guanylate cyclase activator (i.e., BAY 60–2770) to these rats improved their AVF remodeling^34^. In the NOS3 front, Croatt *et al*. reported that the administration of NG-nitro-L-arginine methyl ester (L-NAME), a global NOS activity inhibitor non-specific to NOS3, impaired AVF remodeling in rats with normal kidney function^36^. The present study uses genetically modified mice that overexpress NOS3 (gain-of-function) or do not express NOS3 (loss-of-function) to establish the causal relationship between NOS3 and AVF remodeling. To the best of our knowledge, the present study is the first to provide solid evidence that NOS3 is a critical molecule driving successful AVF remodeling. This is the first innovation of our study.

The second innovation is that our study presents the first FSI study that offers detailed characterization of both hemodynamics and wall mechanics in murine AVFs. It has long been postulated that aberrant vascular mechanics may lead to pathological AVF remodeling, using the analogy that pathological arterial mechanics causes pathological arterial remodeling. For example, atherosclerosis preferentially occurs in regions of arterial branches with disturbed flow, but not in relatively straight arteries where blood flow is usually laminar^31,32,37^. Furthermore, in tissues, increased blood pressure^38,39^ and wall stretch^39,40^ have been shown to induce arterial wall thickening; at the cellular level, wall stretch has been shown to stimulate the proliferation of arterial and venous SMCs^41–43^. In the field of AVF research, several experimental and clinical studies have been performed to investigate the association between hemodynamics and AVF maturation^44–51^. However, previous studies usually have limitations such as a temporal and spatial resolution of 10 ms and 300 μm for the fluid domain^46^, assume the wall to be rigid, and only consider FSS at the wall (i.e., τ_w_)^46,48^. Furthermore, the limited published FSI studies have lower resolution than ours^52–54^ and are focused on comparing the τ_w_ values derived from rigid- vs. deformable-wall assumptions^53,54^. Our FSI study of the murine AVFs has brought several advancements to this field of research. Our temporal resolution is 0.1 ms, our spatial resolution is 0.4 μm for the fluid domain and 0.33 μm for the solid domain, and our solid domain is composed of three layers rather than the more commonly used monolayer^50,52^ in order to improve the accuracy of simulation results. We also more comprehensively consider the vascular mechanics, presenting velocity, Reynolds number, wall hemodynamic parameters (i.e., τ_w_ and OSI), luminal FSS, volumetric hemodynamic parameters (i.e., vorticity, relative helicity, Q-criterion) and wall deformation (i.e., inner wall circumferential stretch and radial wall thinning). We found that smoother blood flow streamlines and lower τ_w_, blood vorticity, inner wall circumferential stretch, and radial wall thinning are associated with favorable AVFs (i.e., larger AVF lumen and smaller NH).

A third innovation of our study is that the communication between NOS3 and vascular mechanics appears to be two-way. The literature thus far has focused on the effect of hemodynamics on NOS3, and not the opposite direction. The effect of blood flow on the NOS3-NO system has been long well recognized in the arterial system. In 1996, Cooke’s group reported that laminar τ_w_ induced the release of NO from cultured human aortic ECs^55^. Since then, it has been well-established that laminar τ_w_ activates NOS3 and increases its expression, though this effect is not linear. Metaxa *et al*. exposed cultured bovine aortic ECs to laminar τ_w_ ranging from 15 to 100 dyne/cm^2^ and found the expression of NOS3 increased with τ_w_ and reached a plateau at around 40 dyne/cm^2 ^^56^. Furthermore, when compared to laminar τ_w_, disturbed τ_w_ is known to inhibit the expression and/or activation of NOS3 in arterial ECs^57^. Taken together, these previous studies firmly establish the effect of mechanical stresses on NOS3. The current study, however, suggests the effect of NOS3 on mechanical stresses. Specifically, using the combination of genetically modified mice and FSI simulations, we found that overexpression of NOS3 led to smoother blood flow streamlines and lower τ_w_, blood vorticity, inner wall circumferential stretch, and radial wall thinning in the AVF, whereas genetic ablation of NOS3 led to more chaotic blood flow streamlines and higher τ_w_, blood vorticity, inner wall circumferential stretch, and radial wall thinning in the AVF. These results suggest that the expression levels of NOS3 can regulate vascular mechanics, especially in the vein.

Our focus on the vein is yet another innovation of our study. Although the effect of blood flow on the NOS3-NO system is well established in the arterial system, it has not been extensively investigated in the venous system. Arterial structure and function are significantly different from venous structure and function^58–60^. For example, the arterial wall is thicker and has more vascular SMCs than the venous wall^59^. The phenotype of arterial and venous ECs and SMCs are also significantly different^20–22^. For example, the Eph family transmembrane ligand, ephrinB2, is expressed in arterial ECs and it receptor tyrosine kinase, Eph receptor B4 (EphB4), is expressed in venous ECs^21^. Importantly, it has been shown that endothelium-dependent relaxation is greater in the mammary artery than in the saphenous vein^61^. Thus, the influences of NOS3-NO signaling axis may differ between the artery and the vein. While the direct comparison between arteries and veins is beyond the scope of our study, we found that overexpression of NOS3 led for favorable AVF vein’s remodeling, possibly through increasing cGMP to relax smooth muscle and decreasing NH formation to preserve open lumen.

Finally, with regard to clinical applications, our study suggests that strategies to increase the availability of NO directly (e.g., by delivering NO) or indirectly (e.g., by upregulating NOS3 expression and/or activation) may be effective at promoting AVF maturation. Currently AVF maturation failure has no effective treatment in the clinical setting and remains a huge unmet clinical need. Novel therapies directed at targets that can prevent AVF maturation failure is of high clinical significance. Specifically, therapies that can positively modify hemodynamic adaptation and also tailored to improve “vein” adaptation following AVF creation would be novel and significant to improve AVF outcomes. Our study suggests that NO-based pathways may be a potential therapeutic target to modify hemodynamics and biology in the venous environment.

Our study has several strengths. It combines the use of genetically modified animals and an image-based biomechanical modeling method that allows for mechanistic studies of NOS3 biology and vascular mechanics. Previous biomechanical modeling of AVFs has largely used the rigid-wall assumption in CFD simulations^27,28,62–65^. Limited FSI studies have found that velocity and τ_w_ values derived from the rigid-wall assumption in CFD simulations and those from the deformable-wall assumption in FSI are comparable, though the τ_w_ values from rigid-wall simulation are about 10–20% bigger^54,66^, and we have found the same (Supplementary Figs S3 and S4). However, our method has higher temporal and spatial resolution, and we also consider hemodynamic and wall mechanical parameters more comprehensively. Thus, our study offers a more detailed profile of the vascular mechanics in AVFs. In addition, our techniques are positioned to be applied to AVF patients in the clinical setting. Our MRI procedure is contrast agent-free, and thus can be used on AVF patients who are known to be susceptible to the toxicity associated with MRI contrast agents^67,68^. Our MRI-FSI pipeline can also be used to identify regions of potential stenosis, enabling early detection and treatment of AVF stenosis. Current clinical methods typically require a drop in the volumetric flow rate by Doppler ultrasound to identify stenosis, and is the most reliable when stenosis is significant. Our methods offer several improvements, including that it has higher spatial resolution and lower inter-user variability than ultrasound.

The limitations in our study are primarily related to the higher demands of *in vivo* information needed for performing FSI simulations, which introduce additional assumptions for the wall structural parameters and mechanical properties, and additional boundary conditions of the AVF. Here, we assumed a homogenous and linearly elastic artery and vein wall; we also assumed that the boundaries of the AVF (distal and proximal artery, proximal AVF vein) were fixed and did not allow translational or rotational motion during the simulation. Future FSI simulations of AVFs could consider heterogeneous vascular wall thickness and mechanical properties, and relax assumptions at the AVF boundaries, when this information becomes available, so that the simulation domain would more closely resemble the AVF’s natural structural properties, mechanical properties, and behaviors.

In conclusion, when compared to wild-type and NOS3 knockout, overexpression of NOS3 leads to favorable AVF remodeling (i.e., larger AVF lumen and smaller NH), smoother blood flow streamlines, as well as lower fluid shear stress at the wall, blood vorticity, inner wall circumferential stretch, and radial wall thinning. Our results show that NOS3 drives successful AVF remodeling, which may be mediated by the AVF vascular mechanics. These results suggest that enhancing NOS3 expression may be an effective strategy to promote AVF maturation.

## Methods

All animal studies and experiments were approved by the University of Alabama at Birmingham Institutional Animal Care and Use Committee (IACUC) and performed in accordance with National Institutes of Health guidelines.

### Mouse strains and AVF creation surgery

C57BL/6 mice (NOS3+/+) and NOS3 knockout mice (NOS3−/−) on C57BL/6 background were purchased from Jackson Laboratories, Bar Harbor, ME. Transgenic mice overexpressing the human NOS3 gene (NOS3 OE) on C57BL/6 background were described in previous publications^69,70^. The varying NOS3 expression levels in these three mouse strains before AVF creation were confirmed by Western Blot (Supplementary Fig. S5). We have described the procedures required to create AVFs in mice^28^. Briefly, an end (vein) to side (artery) fistula was surgically created between the jugular vein and carotid artery (Supplementary Fig. S1), similar to human venous end-to-arterial side AVFs. We have applied the MRI-FSI pipeline described below on several mice from each strain. We present the results from a representative analysis (n = 1) for each strain in this paper.

### MRI image acquisition

MRI image acquisition was performed using procedures we have described^28^. Briefly, a two-dimensional (2D) time-of-flight (ToF) sequence was used to visualize the global AVF lumen geometry and determine the appropriate and optimal orientations of the remaining scans. Next, a 2D T2-weighted fast spin echo sequence was used with a black-blood (BB) double inversion preparation to visualize the vessel lumens at a higher spatial resolution than 2D ToF imaging; these 2D BB images were used to create the 3D geometry of the AVF fluid domain as described immediately below. Next, a 2D gradient echo velocity mapping sequence was used to obtain the velocity over a cardiac cycle at three locations in the AVF: proximal artery, distal artery, and proximal AVF vein. These velocity profiles were extracted as described in “*AVF blood flow extraction*” and then used both as the velocity boundary conditions of the arteries as well as to calculate the pressure boundary condition of the proximal AVF vein in simulations.

### Lumen segmentation, reconstruction, and meshing to build the AVF lumen geometric model

The vascular lumen was segmented manually from the 2D BB images using Amira 5.2.1 (Visage Imaging, Inc., San Diego, CA), reconstructed to a 3D surface in STL format, and then smoothed in Amira^28^. This smoothed reconstruction was used to create a high-resolution tetrahedral 3D mesh in ANSYS ICEM 15.0 (ANSYS, Inc., Canonsburg, PA). 1.5 × 10^6^ tetrahedra in the lumen (i.e., the fluid domain) were chosen for all simulations based on the mesh independence we performed^28^.

### AVF blood flow extraction

Blood flow velocity over a cardiac cycle was extracted from 2D gradient echo velocity mapping scans using Segment 1.9 (Medviso SB, Lund, Sweden).

### CFD modeling of the fluid domain (i.e., the blood flow) in FSI simulations

The fluid domain of our AVF lumen geometric model was simulated using a procedure that was modified significantly from that described previously by us which assumed the vessel wall to be *rigid*^28^; several modifications were made in the present study which assumes the vessel wall to be *deformable*. First, while the proximal and distal artery still used velocity boundary conditions for CFD modeling as previously described^28^, the boundary condition for the proximal AVF vein is now prescribed as a pressure waveform calculated from the two-element R-C Windkessel model. This model uses the measured blood flow in the proximal artery, lumped downstream vascular resistance (R) and compliance (C) to estimate a pulsatile pressure waveform in blood flow^71^. Using pressure values from the literature for mice^72,73^, we estimated R and C values of the downstream proximal AVF vein for each individual mouse. Second, for coupling with the solid domain in FSI simulations, the Navier-Stokes equations were expressed in arbitrary Lagrangian-Eulerian (ALE) form^53^. The blood properties (assumed to be incompressible and Newtonian) and the temporal discretization strategies (time step = 0.1 ms; approximately 800–1200 time steps over the cardiac cycle) for the pulsatile blood flow were as described by us^28^.

### Finite element modeling of the structural domain (i.e., the vessel wall) in FSI simulations

The structural domain of the vessel wall was modeled using discrete-Kirchhoff-theory-based shell finite elements^53^, which extruded outwards from the outermost fluid mesh layer into the solid domain consisting of three mesh layers. We used three layers instead of the more commonly used single mesh layer^53,54^ in order to improve the accuracy of simulation results. The AVF wall mesh contains a total of approximately 0.3 million elements. The wall thickness was 0.01 and 0.005 mm for the mouse artery and vein, respectively^74,75^. Young’s modulus and Poisson’s ratios were defined as 120 kPa and 0.4 respectively for the mouse artery, and 80 kPa and 0.4 for the mouse vein^76,77^. Two-way transient FSI was performed in ANSYS Workbench, implicitly coupling the blood flow simulation in ANSYS Fluent to the vessel wall deformation in ANSYS Mechanical. The FSI boundary conditions were (i) equal displacement of the fluid and solid domains and (ii) equilibrium of forces at the fluid-solid interface. Convergence criteria was set at 1 × 10^−5^ for all residuals.

### Post-processing

To investigate the vascular mechanics of the AVF, we calculated the *hemodynamic* parameters and the *wall mechanical* parameters, using Tecplot 360 (Tecplot, Inc., Bellevue, WA). The *hemodynamic* parameters include both wall and volumetric components. *Wall hemodynamic parameters* include: (i) the magnitude of fluid shear stress (FSS, Equation 1; τ = shear stress; x, y, z = coordinates) at the wall (τ_w,_ Equation 2; w = wall), which is the frictional force at the blood-vessel wall interface, and (ii) the oscillatory shear index at the wall (OSI, Equation 3; t = time in the cardiac cycle), which is the magnitude of change in the blood flow direction over a cardiac cycle and it value ranges between 0 and 0.5, with 0 indicating no change and 0.5 indicating full directional reversal. *Volumetric hemodynamic parameters* include: (i) vorticity (Ω, Equation 4; u = the blood velocity vector), which is the magnitude of blood rotation with no directional component^78^; (ii) relative helicity (Equation 5), which is a normalized measurement of the direction of blood rotation ranging from +1 (completely clockwise) to −1 (completely counterclockwise)^79^; and (iii) Q-criterion (Equation 6; S = the shear strain rate), which is a measurement of the dominance of parallel blood flow (e.g., in the direction of bulk flow) or rotational blood flow; positive Q-criterion indicates dominant rotational blood flow and a possible vortex, negative Q-criterion indicates dominant parallel flow with little rotation and no vortex, and zero Q-criterion indicates equal magnitudes of rotational and parallel blood flow^80^. The *wall mechanical parameters* include inner wall circumferential stretch (Equation 7) and radial wall thinning (Equation 8). Finally, the Reynolds number is a unitless measure of flow turbulence and defined as |u|D/ν, where |u| = the blood velocity magnitude, D = diameter of the vessel at any cross-section, and ν = blood kinematic viscosity.

The lumen centerline vectors and the tangential vectors to the centerline for each vessel were calculated using Vascular Modeling Toolkit (VMTK); these were then used to determine circumferential slices tangential to the centerline. Mean ± standard deviation (SD) of the parameters (listed above) in these cross-sectional slices were calculated at the distal artery, AVF anastomosis, proximal artery, and proximal AVF vein, every 50 μm. τ_w_ and OSI were calculated at each mesh node of the lumen wall (i.e., the outermost layer of the fluid domain, which is also the innermost layer of the solid domain). Vorticity, relative helicity, and Q-criterion were calculated at each mesh node of the lumen volume. Inner wall circumferential stretch was calculated in cross-sectional slices of the lumen wall (i.e., the innermost layer of the solid domain) perpendicular to the lumen centerline; the baseline circumference was the minimum circumference over the cardiac cycle. Radial wall thinning was calculated at each mesh node of the lumen wall, in cross-sectional slices perpendicular to the lumen centerline; the baseline wall thickness was the maximum wall thickness over the cardiac cycle. Reynolds number was calculated in cross-sectional slices of the lumen volume. All parameters (except OSI) were averaged over a cardiac cycle unless otherwise mentioned.

1 $${FSS}={({\tau }_{x}^{2}+{\tau }_{y}^{2}+{\tau }_{z}^{2})}^{\frac{1}{2}}$$

2 $${FSS}\,{at}\,{the}\,{wall}\,({\tau }_{w})={({\tau }_{w,x}^{2}+{\tau }_{w,y}^{2}+{\tau }_{w,z}^{2})}^{\frac{1}{2}}$$

3 $${OSI}=0.5(1-\frac{|{\int }_{0}^{t}{\tau }_{w}dt|}{{\int }_{0}^{t}|{\tau }_{w}|dt})$$

4 $${Vorticity}\,({\rm{\Omega }})=\nabla \times u$$

5 $${Relative}\,{helicity}=\frac{u\cdot {\rm{\Omega }}}{|u||{\rm{\Omega }}|}$$

6 $${Q}-criterion=\frac{1}{2}{({\rm{\Omega }}}^{2}-{{\rm{S}}}^{2})\,$$

7 $$Inner\,wall\,circumferential\,stretch\,( \% )=100\ast [\frac{(circumference-circumferenc{e}_{baseline})}{circumferenc{e}_{baseline}}]$$

8 $${Radial}\,{wall}\,{thinking}\,(\mu m)=wall\,thicknes{s}_{baseline}-wall\,thickness$$

### cGMP analysis from AVF veins

Blood vessels were snap frozen at the time of tissue harvest and then analyzed for cGMP using a commercial enzyme immunoassay kit (GenScript®, Piscataway, NJ) according to the manufacturers’ instructions. The levels of cGMP were expressed as pmol/mg protein.

### Morphometric analysis to obtain intima/media area ratios

Formalin-fixed and paraffin-embedded AVF samples were cut into thin sections. 10–12 slides of 5 μm sections were obtained by selecting the first of every 10 sections beginning at the venous side of the AVF anastomosis. Morphometric analysis was performed by using Russell-Movat pentachrome-stained slides. Briefly, digital photographs of each tissue section were taken at final magnification of 10X. Intimal and medial regions were manually outlined and tissue areas were measured by using Cellsense Dimension Software (Olympus Life Science). The ratio of intimal area to medial area was calculated for each tissue section and mean value for the intimal to medial area ratio was reported for each animal.

### Statistical methods

Group comparisons of simulation results were performed in GraphPad Prism 6 (GraphPad Software, Inc., La Jolla, CA) by paired t-test (if data followed a normal distribution) or Wilcoxon signed-rank test (if data did not follow a normal distribution), with significance set at p < 0.05. The levels of cGMP and the intima/media area ratios were compared using ANOVA, with significance set at p < 0.05.

## Supplementary information

SUPPLEMENTARY FIGURES

## Data Availability

The datasets generated during and/or analyzed during the current study are available from the corresponding author on reasonable request.
