# Author Correction: The effect of endothelial nitric oxide synthase on the hemodynamics and wall mechanics in murine arteriovenous fistulas

**DOI:** 10.1038/s41598-019-51080-5

**Published:** 2019-10-24

**Authors:** Daniel Pike, Yan-Ting Shiu, Yun-Fang Cho, Ha Le, Maheshika Somarathna, Tatyana Isayeva, Lingling Guo, J. David Symons, Christopher G. Kevil, John Totenhagen, Timmy Lee

**Affiliations:** 1 Department of Biomedical Engineering, University of Utah, Salt Lake City, UT USA; 2 Division of Nephrology and Hypertension, Department of Internal Medicine, University of Utah, Salt Lake City, UT USA; 3 Veterans Affairs Medical Center, Salt Lake City, UT USA; 4 Department of Medicine and Division of Nephrology, University of Alabama at Birmingham, Birmingham, AL USA; 5 Department of Nutrition and Integrative Physiology and Molecular Medicine Program, University of Utah, Salt Lake City, UT USA; 6 Division of Endocrinology, Metabolism, and Diabetes, University of Utah, Salt Lake City, UT USA; 7 Departments of Pathology, Molecular and Cellular Physiology, and Cellular Biology and Anatomy, LSU Health Shreveport, Shreveport, LA USA; 8 Department of Radiology, University of Alabama at Birmingham, Birmingham, AL USA; 9 Veterans Affairs Medical Center, Birmingham, AL USA

Correction to: *Scientific Reports* 10.1038/s41598-019-40683-7, published online 12 March 2019

This Article contains an error in the Methods section under subheading ‘AVF blood flow extraction’ where,

“AVF blood flow extraction

Blood flow velocity over a cardiac cycle was extracted from 2D gradient echo velocity mapping scans using Segment 1.9 (Medviso SB, Lund, Sweden).”

should read:

“AVF blood flow extraction

Blood flow velocity over a cardiac cycle was extracted from 2D gradient echo velocity mapping scans using ImageJ (U.S. NIH, Bethesda, MD).”

