# Supplementary material for: The effect of endothelial nitric oxide synthase on the hemodynamics and wall mechanics in murine arteriovenous fistulas
Source: Sci Rep. 2019 Mar 12;9:4299. doi: 10.1038/s41598-019-40683-7 (PMC6414641; doi:10.1038/s41598-019-40683-7)
Supplement: Supplementary file 1 — SUPPLEMENTARY FIGURES [file 41598_2019_40683_MOESM1_ESM.docx]

**SUPPLEMENTARY FIGURES**

**The effect of endothelial nitric oxide synthase on the hemodynamics and wall mechanics in murine arteriovenous fistulas**

Daniel Pike^1,2^, Yan-Ting Shiu^2^, Yun-Fang Cho^2^, Ha Le^2^, Maheshika Somarathna^3^, Tatyana Isayeva^3^, Lingling Guo^3^, J. David Symons^4,5^, Christopher G. Kevil^6^, John Totenhagen^7^, and Timmy Lee^3,8^

Affiliations:

^1^Department of Biomedical Engineering, University of Utah, Salt Lake City, UT

^2^Division of Nephrology and Hypertension, Department of Internal Medicine, University of Utah, Salt Lake City, UT

^3^Department of Medicine and Division of Nephrology, University of Alabama at Birmingham, AL

^4^Department of Nutrition and Integrative Physiology and Molecular Medicine Program, University of Utah, Salt Lake City, UT

^5^Division of Endocrinology, Metabolism, and Diabetes, University of Utah, Salt Lake City, UT

^6^Departments of Pathology, Molecular and Cellular Physiology, and Cellular Biology and Anatomy, LSU Health Shreveport, Shreveport, LA

^7^Department of Radiology, University of Alabama at Birmingham, AL

^8^Veterans Affairs Medical Center, Birmingham, AL

**
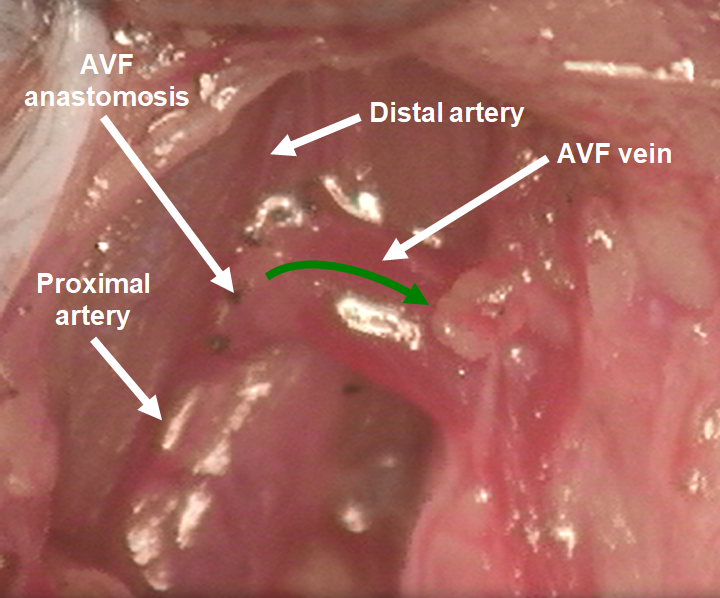
**

**Supplementary Figure S1. Representative mouse AVF model.** Photograph of a representative AVF from a NOS3-/- mouse with end to side anastomosis of jugular vein (end) to carotid artery (side). Green arrow indicates the direction of blood flow in the proximal AVF vein.

**
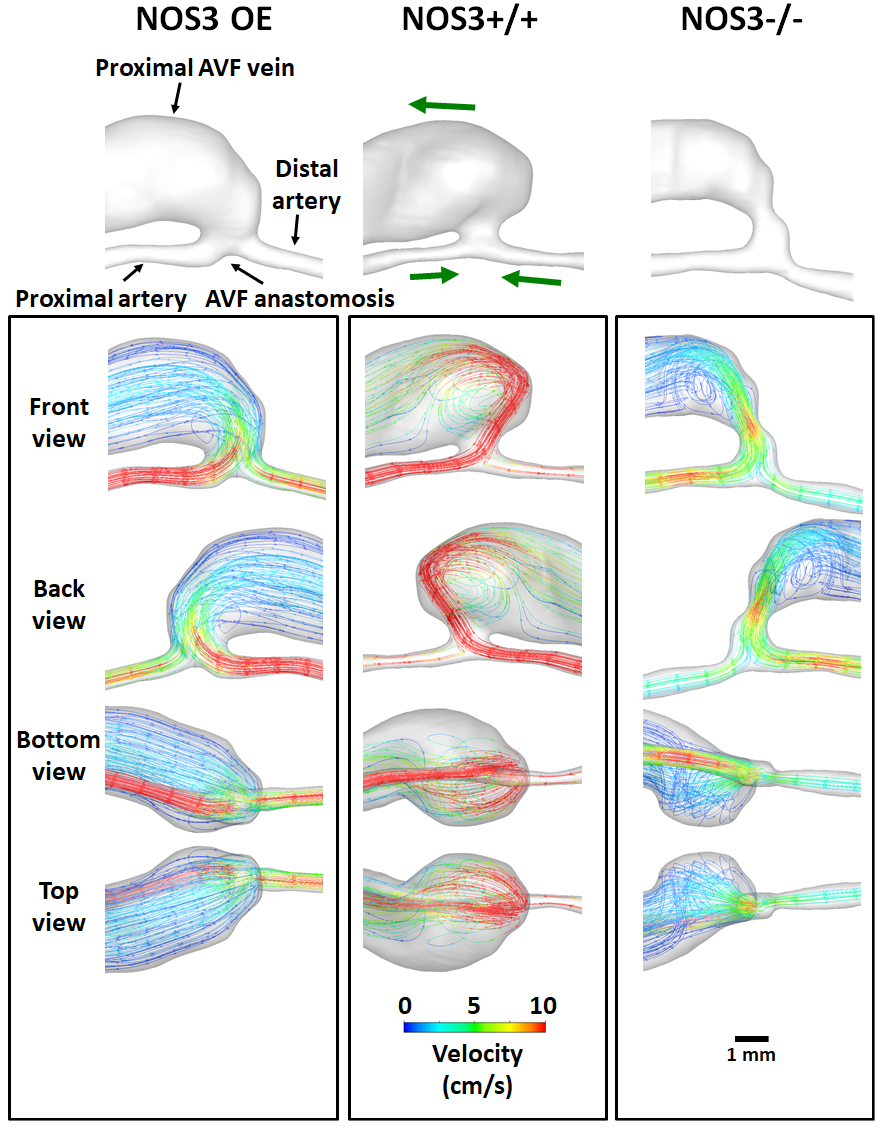
**

**Supplementary Figure S2. Velocity streamlines from the FSI simulations of AVFs in NOS3 OE (left panels), NOS3+/+ (middle panels), and NOS3-/- (right panels) mice.** The labeling of the blood vessels in NOS3 OE and green arrows in NOS3+/+ (which indicate the direction of blood flow) apply to all mice. The velocity color scale and the length scale bar in the bottom apply to all streamline images. Color scales are set to best contrast the differences among the three mice.


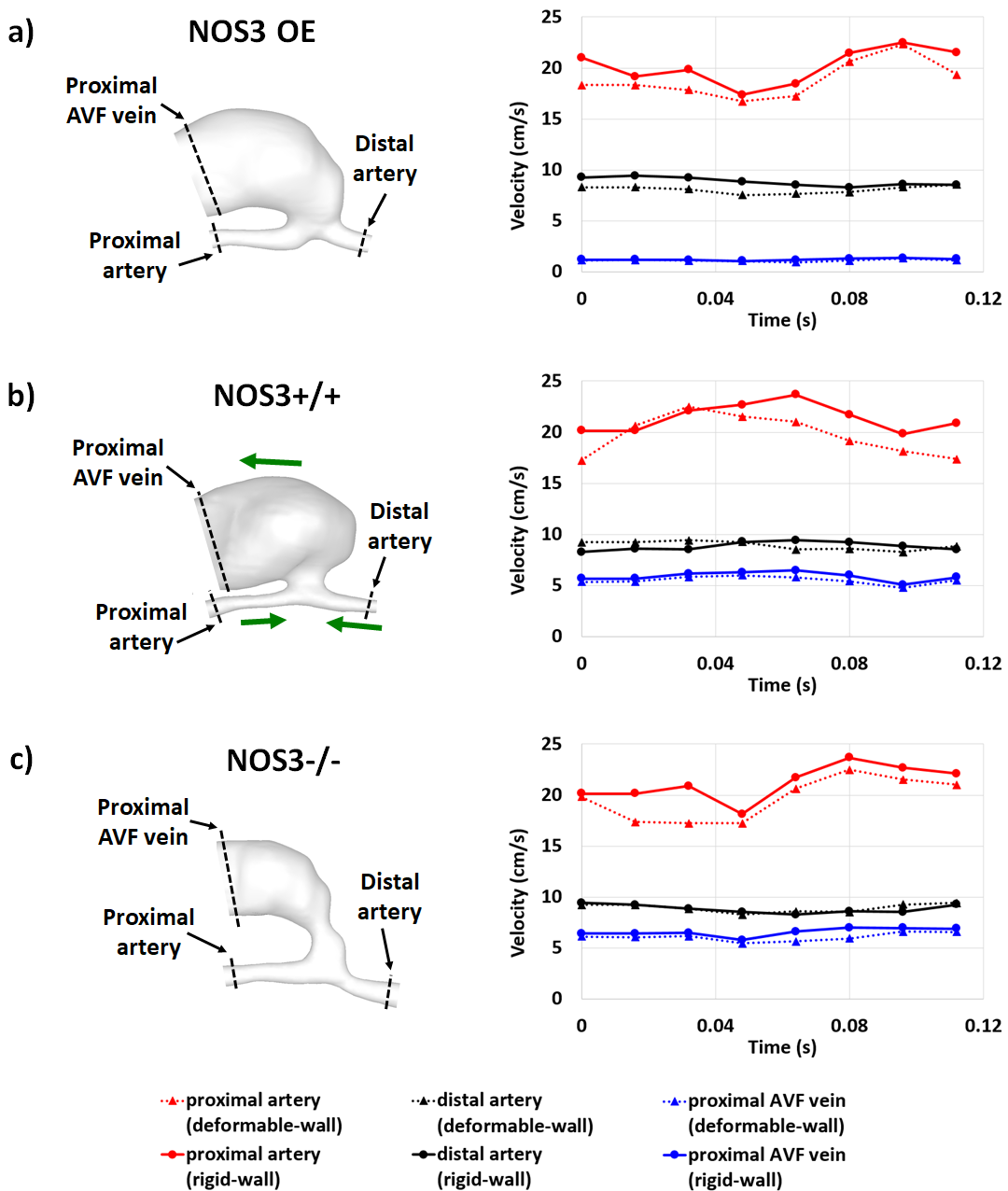


**Supplementary Figure S3. Velocity over the cardiac cycle from the deformable-wall and rigid-wall simulations of AVFs in NOS3 OE (a), NOS3+/+ (b), and NOS3-/- (c) mice.** The green arrows in NOS3+/+ (which indicate the direction of blood flow) apply to all mice. FSI (i.e., deformable-wall) and rigid-wall simulations result in similar velocities over the cardiac cycle at the boundaries (dashed black lines) of the simulation domain.

**
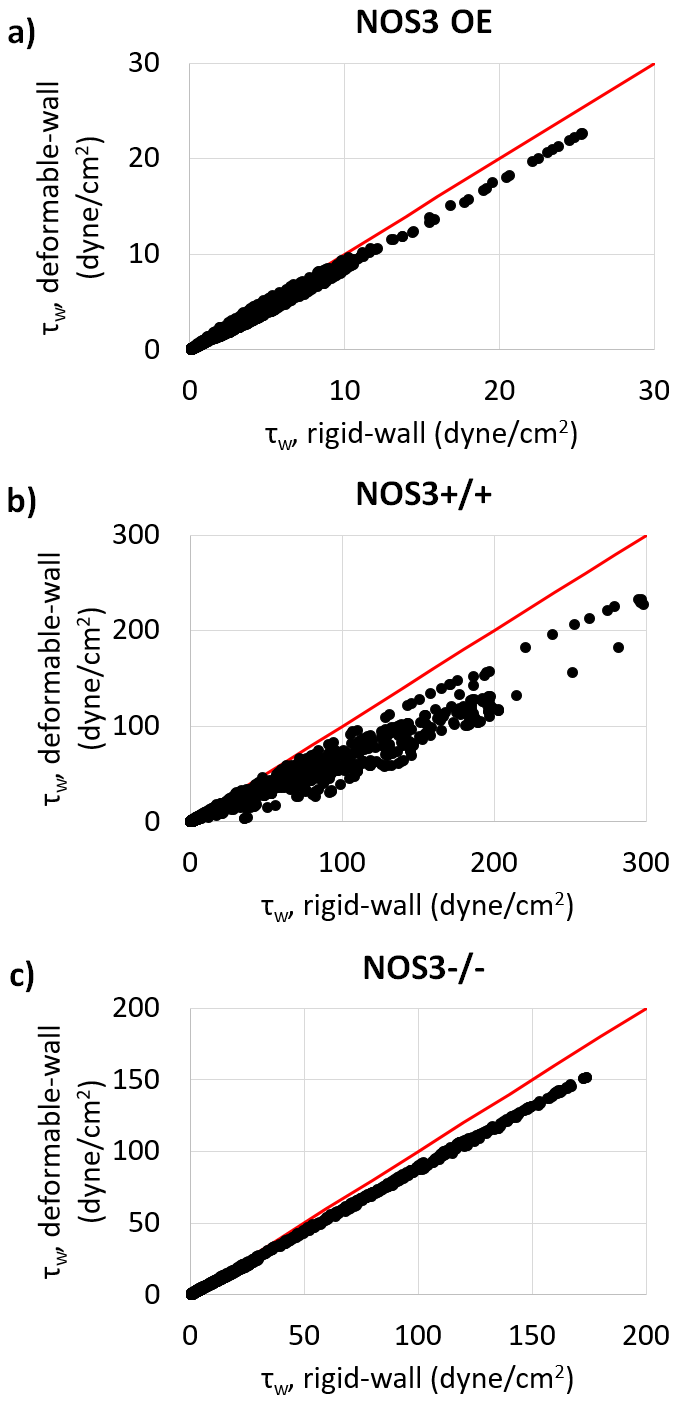
**

**Supplementary Figure S4. Agreement plots of fluid shear stress at the wall (τ_w_) from deformable-wall and rigid-wall simulations of AVFs in NOS3 OE (a), NOS3+/+ (b), and NOS3-/- (c) mice.** Each individual black dot represents the τ_w_ values at each cross-sectional slice, averaged around the circumference and over a cardiac cycle. These slices are in the first 7 mm of the proximal AVF vein starting from the anastomosis (140 slices, with 50 μm between 2 slices) in the lumen geometrical models shown in Fig. 1 (j), (k), and (l). The solid red line is the identity line, indicating where τ_w_ values from deformable-wall and rigid-wall simulations are identical. The black dots in all plots are either near the identity line (when τ_w_ values are small) or below the identity line (when τ_w_ values are large).

**
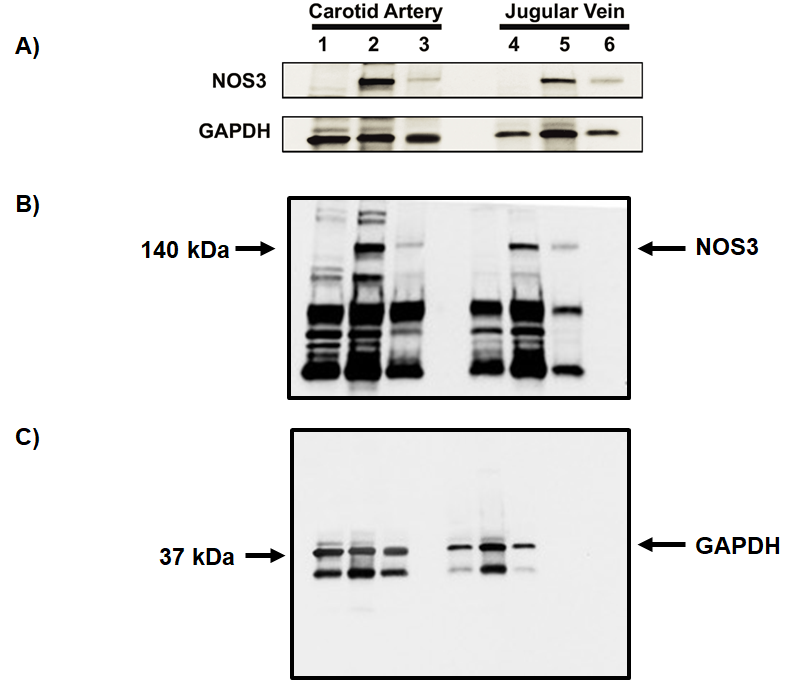
**

**Supplementary Figure S5. Western blot of NOS3 in three mouse strains**. (A) A representative western blot of NOS3 protein in the carotid artery (1-3) and jugular vein (4-6) from NOS3-/- (1,4), NOS3 OE (2,5), and NOS3+/+ (3,6) mice, without AVF creation surgery. All bands for NOS3 are from one gel (B) and all bands for GAPDH are from another gel (C). Antibodies against these specific molecules were used: (1) eNOS/NOS type III (140 kDa), BD Biosciences (San Jose, CA), Catalog No: 610296; and (2) GAPDH (14C10) (37kDa), Cell Signaling Technology (Danvers, MA), Catalog #2118.
